# A CT-based radiomics nomogram for prediction of lung adenocarcinomas and granulomatous lesions in patient with solitary sub-centimeter solid nodules

**DOI:** 10.1186/s40644-020-00320-3

**Published:** 2020-07-08

**Authors:** Xiangmeng Chen, Bao Feng, Yehang Chen, Kunfeng Liu, Kunwei Li, Xiaobei Duan, Yixiu Hao, Enming Cui, Zhuangsheng Liu, Chaotong Zhang, Wansheng Long, Xueguo Liu

**Affiliations:** 1 Department of Radiology, Jiangmen Central Hospital, 23#, North Road, Pengjiang Zone, Jiangmen, Guangdong Province 529030 People’s Republic of China; 2 School of electronic information and automation, Guilin University of Aerospace Technology, Guilin City, Guangxi Province 541004 People’s Republic of China; 3 Department of Radiology, The Fifth Affiliated Hospital of Sun Yat-sen University, Zhuhai, Guangdong Province 519000 People’s Republic of China; 4 Department of Nuclear Medicine, Jiangmen Central Hospital, Jiangmen, Guangdong Province 529030 People’s Republic of China; 5 Department of Radiology, Guangzhou First People’s Hospital, Guangzhou City, Guangdong Province 510180 People’s Republic of China

**Keywords:** Computed tomography, Lung adenocarcinoma, Solitary pulmonary nodule, Sub-centimeter

## Abstract

**Purpose:**

To develop a radiomics nomogram based on computed tomography (CT) images that can help differentiate lung adenocarcinomas and granulomatous lesions appearing as sub-centimeter solid nodules (SCSNs).

**Materials and methods:**

The records of 214 consecutive patients with SCSNs that were surgically resected and histologically confirmed as lung adenocarcinomas (*n* = 112) and granulomatous lesions (*n* = 102) from 2 medical institutions between October 2011 and June 2019 were retrospectively analyzed. Patients from center 1 ware enrolled as training cohort (*n* = 150) and patients from center 2 were included as external validation cohort (*n* = 64), respectively. Radiomics features were extracted from non-contrast chest CT images preoperatively. The least absolute shrinkage and selection operator (LASSO) regression model was used for radiomics feature extraction and radiomics signature construction. Clinical characteristics, subjective CT findings, and radiomics signature were used to develop a predictive radiomics nomogram. The performance was examined by assessment of the area under the receiver operating characteristic curve (AUC).

**Results:**

Lung adenocarcinoma was significantly associated with an irregular margin and lobulated shape in the training set (*p* = 0.001, < 0.001) and external validation set (*p* = 0.016, = 0.018), respectively. The radiomics signature consisting of 22 features was significantly associated with lung adenocarcinomas of SCSNs (*p* < 0.001). The radiomics nomogram incorporated the radiomics signature, gender and lobulated shape. The AUCs of combined model in the training and external validation dataset were 0.885 (95% confidence interval [CI]: 0.823–0.931), 0.808 (95% CI: 0.690–0.896), respectively. Decision curve analysis (DCA) demonstrated that the radiomics nomogram was clinically useful.

**Conclusion:**

A radiomics signature based on non-enhanced CT has the potential to differentiate between lung adenocarcinomas and granulomatous lesions. The radiomics nomogram incorporating the radiomics signature and subjective findings may facilitate the individualized, preoperative treatment in patients with SCSNs.

## Background

Computed tomography (CT) can demonstrate small lung nodules that are invisible on chest radiographs. Lung nodules are classified into 3 subtypes as non-solid, part-solid and solid according to their attenuation on CT images [1]. Most lung sub-centimeter solid nodules (SCSNs) are benign, and approximately 80% are granulomas [2]. On the other hand, lung adenocarcinoma is the most common histological type of peripheral lung cancer, and its incidence has been increasing in recent years [3].

Once identified, pulmonary SCSNs must be evaluated to determine the likelihood of malignancy, and to determine management recommendations. The lung imaging reporting and data system (Lung-RADS) is a risk-stratifying classification system for the results of low-dose chest CT performed for lung cancer screening, and the standard recommendation has been to closely follow-up SCSNs at frequent intervals (3 to 12 months) based on nodule size and growth pattern [4]. However, this recommendation increases health care costs, results in substantial radiation exposure, and imposes psychological stress upon individuals [5]. As such, different imaging methods have been studied to distinguish malignant from benign SCSNs in order to facilitate earlier diagnosis and treatment [6–8]. Studies have indicated that SCSNs with a larger size, lobulated or spiculated morphology, and irregular margin were more likely to be malignant [6, 8]. However, inter-reader variability with respect to manual nodule size measurement and visual assessment of radiologic features has been reported, which could lead to misdiagnoses [9, 10]. Meanwhile, SCSNs remains a diagnostic challenge in ^18^F-labeled fluoro-2-deoxyglucose positron emission tomography (^18^F-FDG PET/CT) because they are beyond the resolution of PET/CT [11, 12]. Several studies have reported a relatively lower diagnostic accuracy for smaller lesions in CT-guided percutaneous fine-needle aspiration biopsy (FNAB), ranging from 52 to 88% [13, 14].

Radiomics is the process of converting medical imaging data to quantitative, mineable features through advanced computational methodologies, which can be used to develop decision systems to accurately estimate patient risk and improve individualize treatment [15, 16]. Studies have shown that radiomics features extracted from chest CT images can be used for predicting lung nodule malignancy [17], differentiating histological subtype [18], determining gene expression [19], and evaluating post-treatment prognosis [20]. A few investigators have attempted to distinguish granulomas from malignancies using quantitative radiomics, or computerized feature-based analysis [21–23]. However, these studies were limited by small sample size, incomplete normalized enrollment criteria, and the results were not validated based on multicenter data sets.

Thus, the purpose of this study was to determine if radiomics nomogram based on non-enhanced chest CT images can distinguish primary lung adenocarcinomas from granulomatous lesions in patients with peripheral SCSNs. Furthermore, we collected datasets from 2 independent hospitals, and all methods were independently evaluated in external dataset.

## Methods

### Patient selection

This retrospective study was approved by the Ethical Review Boards of the 2 participating hospitals. Because of the retrospective nature of the study, the requirement of patient informed consent was waived. We retrospectively reviewed the medical records of all patients who had undergone surgical resection for lung adenocarcinomas and granulomatous lesions that were identified as peripheral SCSNs on chest CT images between October 2011 and June 2019. Criteria for inclusion in the analysis were: 1) Histopathologically confirmed primary lung adenocarcinomas or granulomatous lesions of the surgical resection tissue specimens; 2) Solitary solid peripheral lung nodule ≤ 10 mm in diameter; 3) Preoperative chest CT images with a thin slice thickness (≤ 1.5 mm); 4) Interval between preoperative chest CT scan and surgery less than 2 weeks. Exclusion criteria were: 1) Solitary sub-solid nodules (non-solid and part-solid); 2) Obvious calcifications or satellite opacities in the lung nodule; 3) Pathologic diagnosis by examination of a biopsy tissue specimen, or bronchoscopy; 4) Chest CT images with artifacts and/or not of sufficient quality for diagnosis; 5) Patients with a previous medical history of a malignant tumor.

**Fig. 1:**
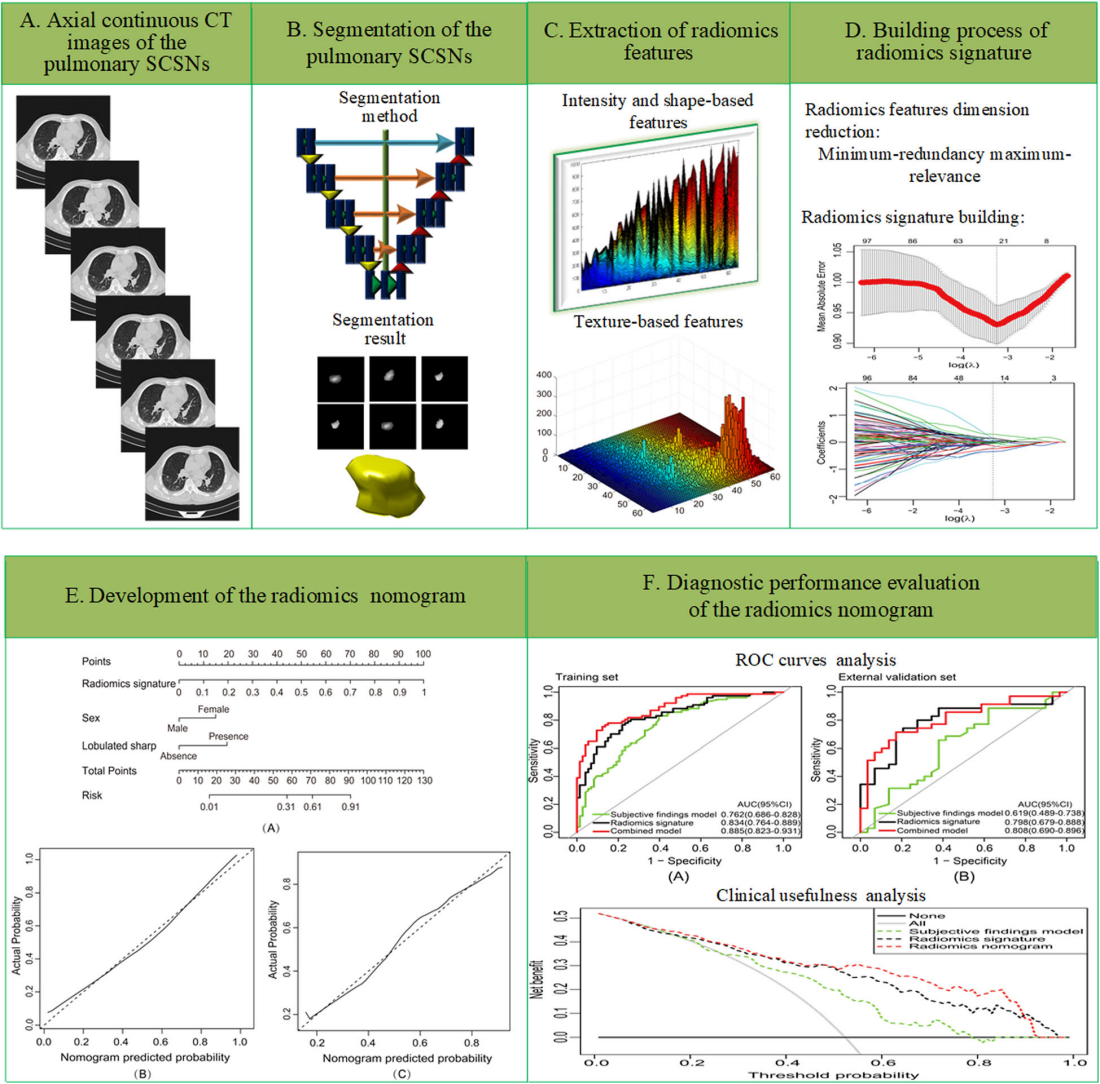
Overview of the study methodology

A total of 150 consecutive patients (83 males and 67 females; mean age, 55.45 ± 12.26 years; age range, 20–81 years) from Center 1 were enrolled as training dataset, with 77 lung adenocarcinomas and 73 granulomatous lesions. The independent external validation dataset consisted of 64 consecutive patients from Center 2 (31 males and 33 females; mean age, 56.09 ± 11.36 years; age range, 29–78 years), with 35 lung adenocarcinomas and 29 granulomatous lesions. An overview of the study methodology is illustrated in Fig. 1.

### Chest CT scan technique

All chest CT examinations were included the entire thorax, and were performed with supine position. Single scans were obtained during deep inspiration and breath-hold. CT scans were performed with Somatom Sensation 16-detector and Dual-energy Force (Siemens Medical System, Forchheim, Germany), Toshiba Aquilion 64-slice CT scanner (Toshiba Medical Systems, Japan), or GE Discovery CT750 64-detector CT scanner (GE Medical Healthcare, Milwaukee, Wisconsin). Scanning parameters were: 120 kVp; 40–80 mAs with auto exposure control; pitch 0.875–1.5; detector collimation 0.625–2.5 mm; field of view (FOV) 360 mm X 360 mm. Lung images were reconstructed with the use of a high-spatial-frequency algorithm, and mediastinal images with the use of an intermediate-spatial-frequency algorithm. Contiguous images were reconstructed with a 0.625–1.5 mm slice thickness for axial sections, and a 3.0 mm slice thickness for coronal and sagittal sections.

### Chest CT image evaluation

CT images were reviewed using a picture archiving and communication system (PACS). The images were read using a lung window of − 550 Hounsfield units (HU) and width of 1500 HU, and a mediastinal window of 35 HU and a width of 250 HU. Two experienced radiologists with subspecialty training in thoracic radiology (reader 1 with more than 15 years’ experience, and reader 2 with 25 years’ experience) who were unware of the final pathological diagnosis and clinical data reviewed the CT scan images of each nodule independently. Nodule characteristics recorded included: 1) Location; 2) Size; (3) Margin (regular, irregular); (4) Lobulated shape (absent, present); 5) Spiculated sign (absent, present). Nodule size was determined by the average of the maximum longest diameter and perpendicular short diameter on the axial CT images on which the nodule was the largest. A regular margin was defined as sharply demarcated, with a round or oval smooth shape. Lobulated shape was defined as a part of the nodule surface (except the portions in contact with the pleura) exhibiting a concave or straightened configuration. Spiculated sign was defined as the presence of 2-mm or thicker strands extending from the nodule margin into the lung parenchyma, without reaching the pleural surface [1, 5, 7]. Discrepancies in interpretation between the observers were resolved by consensus.

Gender, age and subjective CT features were compared between groups with the Wilcoxon Rank Sum test or Pearson chi-squared test, as appropriate. Univariate and multivariate logistic regression analyses were conducted. Clinical factors (including gender and age) and subjective CT features which were significantly different between groups on univariate analysis were selected and examined by multivariate logistic regression to develop the predictive subjective findings model.

### Histopathological analysis

Histopathological examinations of the surgical specimens were performed by 2 pathologists with subspecialty training in chest pathological diagnosis (one with more than 10 years’ experience, and the other with 15 years’ experience) who were blinded to the chest CT reports and clinical information. Resected lesions were classified according to the 2011 International Association for the Study of Lung Cancer/American Thoracic Society/ European Respiratory Society classification system, and the 2015 World Health Organization (WHO) classification of lung neoplasms [4, 24].

### Nodule segmentation and radiomics feature extraction

A U-net-based deep learning model was used for volume of interest (VOI) segmentation, and was primarily implemented with Python 2.7 [25]. When inputting the whole slice tumor image into the U-net based deep learning model, the boundaries of the lesions were automatically determined without any pre- or post-processing (**Supplementary****A0**). The whole tumor volume was then reconstructed on multiple 2-dimensional (2D) image slices by interpolation [26]. During the reconstruction of the whole volume lesions, wavelet band-pass filtering, isotropic resampling, and grayscale discretization were performed to obtain heterogeneity parameters of different characteristics, and thus improve the robustness and reproducibility of the extracted heterogeneity parameters [27–29].

Radiomics features were divided into 3 categories: 1) First order features; 2) Intensity and shape-based features; 3) Texture-based features. In total, there were 10, 329 radiomics features. Inter-correlation coefficients (ICCs) were used to assess the reproducibility of the radiomics features. To assess for segmentation variability, one radiologist (Reader 1) randomly selected 30 pulmonary nodules from the training group. Then, 2 in-house segmentation methods derived from a fuzzy speed function-based active counter model (method 2 for 30 lesions) and the U-net-based deep learning model (method 1 for all lesions) were used to obtain VOI 1 and VOI 2 [30, 31]. Then, the radiomics features of the same nodule were extracted from VOI 1 and VOI 2, respectively. The Mann-Whitney U test was used to evaluate each radiomics feature for differentiation of lung adenocarcinomas from granulomatous lesions. The radiomics features with ICC values > 0.75 and significantly different between the lung adenocarcinoma and granulomatous lesion groups were then used in subsequent analyses.

### Radiomics feature selection and radiomics signature model construction

The radiomics features selection and radiomics signature building process were performed in the following 3 steps: 1) radiomics features reproducibility assessment and differences evaluation; 2) reservation of top-ranking features; 3) radiomics signature building with 3 methods. Firstly, radiomics features with ICC values > 0.75 and statistically significant different (*p* < 0.05 in the Mann-Whitney U test) between the lung adenocarcinomas and granulomatous lesions, which were related to lesion heterogeneity, were extracted and standardized by Z-score [32]. Secondly, in the training set, radiomics features were ranked using the minimum redundancy maximum relevance (mRMR) algorithm by maximizing the correlation between radiomics features and SCSNs status, and minimizing the redundancy between radiomics features. In this study, by removing the redundant features, the first 25% highest-ranking features in mRMR were reserved [33]. Thirdly, for radiomics signature building, the least absolute shrinkage and selection operator (LASSO), k-nearest neighbor (KNN) and support vector machine (SVM) were used in the training dataset with 1 × 10-fold nested cross-validation. Respectively. Three radiomics signature models were constructed based on these classifiers, and the model performance was compared through receiver operating characteristic (ROC) curve analysis. Then, the radiomics signature value (Rad-score) of each lesion was calculated using the best radiomics signature model, and the differences of the radiomics features between the lung adenocarcinomas and granulomatous lesion were evaluated using the Mann-Whitney U test.

### Radiomics nomogram construction

A multivariate logistic regression model was constructed using the training set to identify independent factors (including clinical factors, subjective CT features, and radiomics signatures) for differentiating lung adenocarcinomas from granulomatous lesions. A radiomics nomogram was then constructed on the basis of the multivariate logistic regression.

### Performance of the radiomics nomogram in the training and external validation datasets

Nomogram calibration was measured with a calibration curve, and the Hosmer-Lemeshow test was performed to assess the goodness-of-fit of the radiomics nomogram. ROC analysis was performed to evaluate the performance of the radiomics nomogram in the training set and external validation set. The area under the ROC curve (AUC), sensitivity, specificity, accuracy, positive predictive value (PPV), and negative predictive value (NPV) were calculated, respectively. The DeLong test was used to evaluate difference of the ROC curves between various models.

### Clinical value of the radiomics nomogram

To estimate the clinical utility of the nomogram, decision curve analysis (DCA) was performed using all datasets by calculating the net benefits for a range of threshold probabilities [34].

### Statistical analysis

All statistical analyses were performed using R3.0.1 (http://www.rproject.org) and MATLAB software. LASSO was done through the “glmnet” package, ROC analysis and DeLong test were done via “pROC”. The nomogram was completed by “rms”, and DCA was completed by “dca.r.” Multivariable logistic regression was performed with a stepwise backward selection of variables. All AUCs were presented with bootstrap bias-corrected 95% confidence intervals (CIs). All statistical tests were 2-tailed, and values of *p* < 0.05 were considered statistically significant.

## Results

### Clinical characteristics and subjective CT findings of SCSNs

Patient demographic and CT characteristics of the training and validation datasets are presented in Table 1. A total of 214 surgically treated patients (114 males and 100 females; mean age:55.46 ± 12.20 years; age range, 20–81 years) were consecutively enrolled from 2 hospitals. In the lung adenocarcinomas group, 76 nodules were in the upper and middle lobes and 36 were in the lower lobes. In the granulomatous lesions group, 64 nodules were in the upper and middle lobes, and 38 nodules were in the lower lobes. In the training set, there were no differences in the nodule location, size, and spiculated sign between the lung adenocarcinoma and granulomatous lesion groups (*p* = 0.957, 0.357, 0.078, respectively). However, there were significant differences in gender, age, nodule margins and lobulated shape between the 2 groups (*p* = 0.012, 0.006, 0.001, < 0.001, respectively) (Table 1**).** Multivariate analyses revealed gender, age and lobulated shape were independent factors associated with lung adenocarcinomas (odds ratio (OR) = 0.296, 1.043, 4.687, respectively). The AUCs in the training set and external validation set were 0.762 (95% CI: 0.686–0.828) and 0.619 (95% CI: 0.489–0.738), respectively (Table 3**).**

**Table 1:** Clinical characteristics and subjective CT findings of lung adenocarcinomas and granulomatous lesions in in patients with SCSNs

	Training set (*n* = 150)			External validation set(*n* = 64)		
	Lung Adenocarcinomas (77)	Granulomatous Lesions (73)	*P* Value	Lung Adenocarcinomas (35)	Granulomatous Lesions (29)	*P* Value
Gender						
Male	35	48	0.012*	17	14	0.981
Female	42	25		18	15	
Age (mean ± SD, years)	58.44 ± 10.97	52.29 ± 12.82	0.006*	56.60 ± 9.52	55.48 ± 13.41	0.599
Size (mean ± SD, mm)	8.49 ± 1.43	8.41 ± 1.35	0.357	8.78 ± 1.75	8.20 ± 2.71	0.608
Location						
Upper and Middle	52	49	0.957	24	15	0.169
Lower	25	24		11	14	
Margin						
Irregular	56	34	0.001*	26	13	0.016*
Regular	21	39		9	16	
Lobulated sharp						
Absence	30	52	< 0.001*	15	21	0.018*
Presence	47	21		20	8	
Spiculated sign						
Absence	59	64	0.078	29	26	0.676
Presence	18	9		6	3	
Rad-score (mean ± SD)	0.62 ± 0.17	0.40 ± 0.15	< 0.001*	0.58 ± 0.19	0.37 ± 0.16	< 0.001*

Note. The differences were assessed by Wilcoxom Rank Sum test or Pearson χ2 test, as appropriate. *SD*: standard deviation. *p < 0.05. *SCSNs*: sub-centimeter solid nodules

### Radiomics feature selection and radiomics signature model construction

There were 2969 radiomics features with ICC values > 0.75 and that were significantly different between the lung adenocarcinoma and granulomatous lesion groups. Of these, 742 features were selected by the minimum-redundancy maximum-relevance algorithm. The AUCs in the training set of the primary radiomics signature models based on the SVM, KNM, and LASSO classifiers were 0.755 (95% CI: 0.678–0.821), 0.777 (95% CI: 0.702–0.841), and 0.834 (95% CI: 0.764–0.889), respectively. Based on these results, the LASSO method was selected for further radiomics features analysis.

**Fig. 2:**
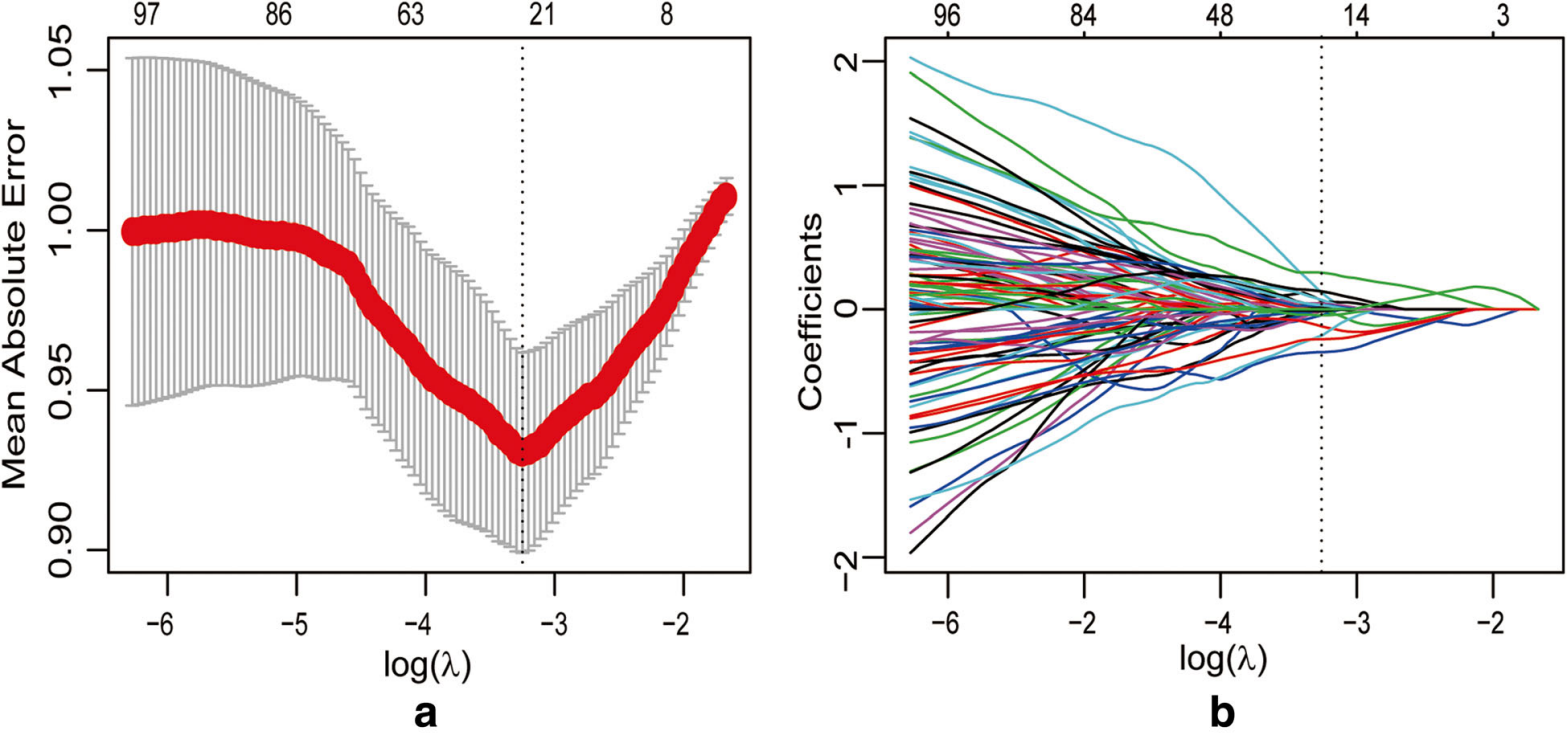
Radiomics feature selection using least absolute shrinkage and selection operator (LASSO) logistic regression. **a** Tuning parameter (λ) selection by 10-fold cross-validation with minimum criteria. Binomial deviance (y-axis) was plotted against log(λ) (x-axis). The dotted vertical lines were drawn at the optimal value of λ, where the model provided the best fit to the data. The optimal value of λ was 0.039, and the corresponding value of log(λ) = − 3.244. **b** LASSO coefficient profiles of the whole features set. The dotted vertical line was plotted at the value selected with 10-fold cross-validation, where 22 optimal features with non-zero coefficients were indicated in the plot

22 radiomics features with non-zero weighted coefficient were saved, and used for building the final radiomics signature model (**Supplementary Table****S1****,** Fig. 2). The AUC for radiomics signature model in the training set was 0.834 (95% CI: 0.764–0.889), and in the external validation set was 0.798 (95% CI: 0.679–0.888).

**Fig. 3:**
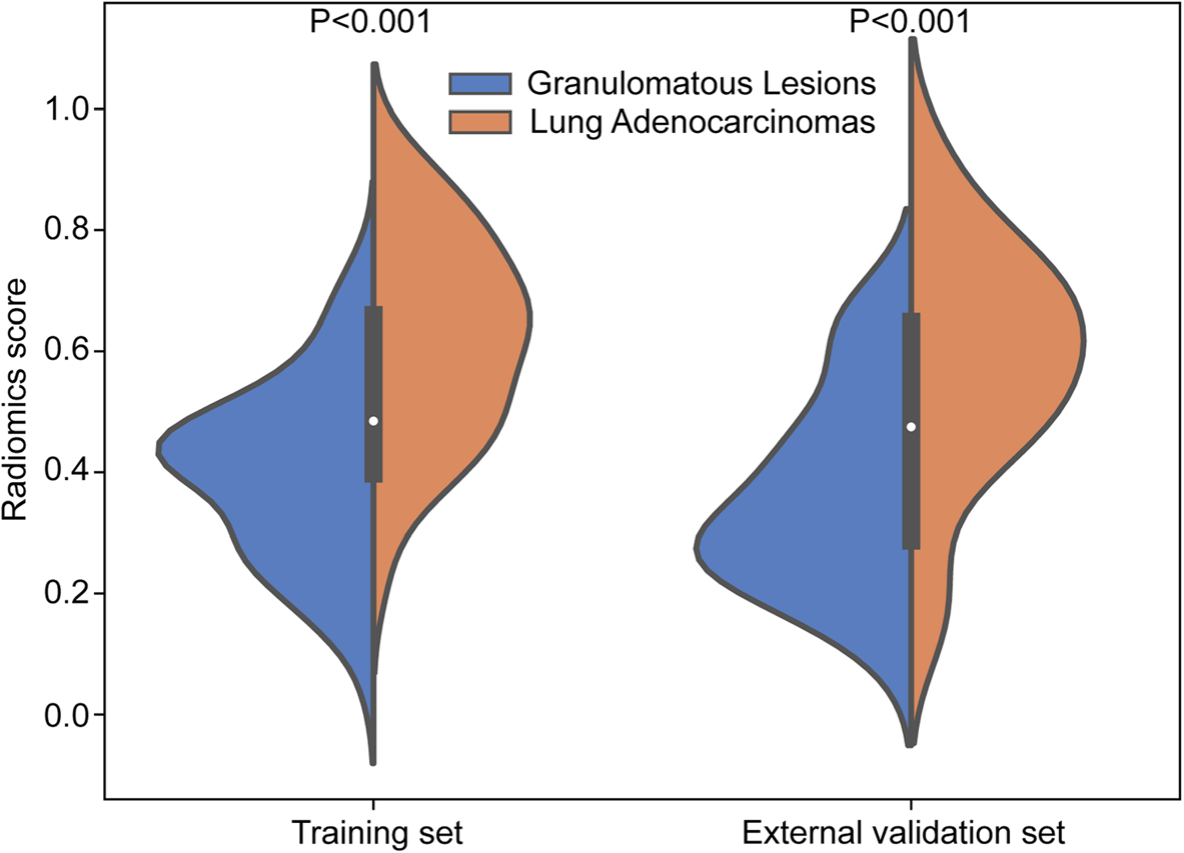
Radiomics score distributions of lung adenocarcinomas and granulomatous lesions in the training dataset and external validation dataset

In the training dataset, the Rad-score of SCSNs in the lung adenocarcinoma group was significantly higher than in the granulomatous lesion group (0.62 ± 0.17 vs. 0.40 ± 0.15; *p* < 0.001). The similar finding was shown in the external validation dataset (0.58 ± 0.19 vs. 0.37 ± 0.16; *p* < 0.001) (Table 1**,** Fig. 3**)**.

**Fig. 4:**
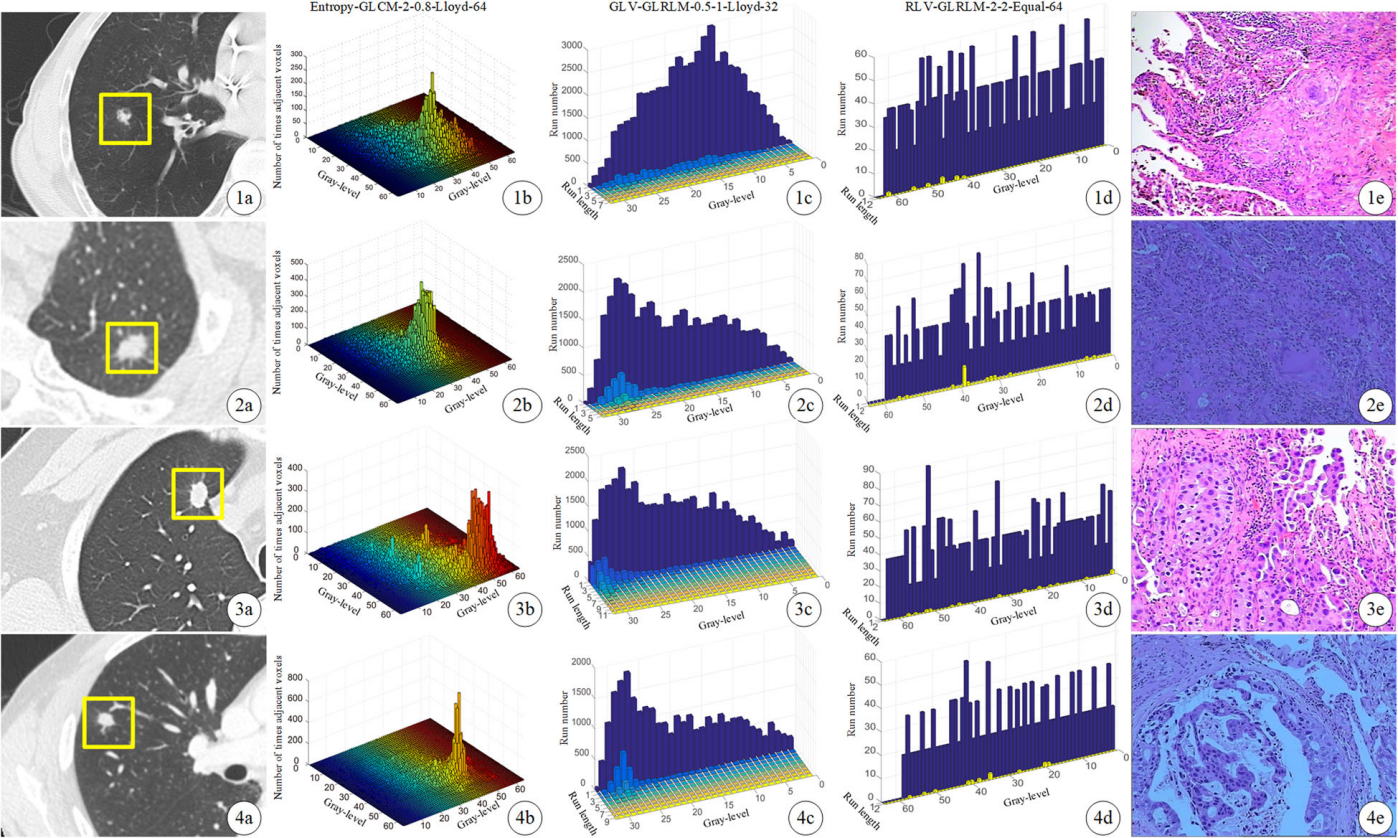
CT images and three selected core radiomics feature maps of patients with pathologic diagnosis. From left to right: (**a**) the original unenhanced axial CT image; (**b**, **c**, **d**) the radiomics feature maps of Entropy-GLCM-2-0.8-Lloyd-64, GLV-GLRLM-0.5-1-Lloyd-32 and RLV-GLRLM-2-2-Equal-64; (**e**) the pathological images (Hematoxylin and Eosin (H&E), × 200). First row: A 55-year-old male with a lung granulomatous lesion in the right lower lobe (nodule size: 8.6 mm; Rad-score: 0.3118). Second row: A 53-year-old female with a granulomatous lesion in the left upper lobe (nodule size: 8.6 mm; Rad-score: 0.4348). Third row: A 52-year-old male with a lung adenocarcinoma in the right upper lobe (nodule size: 8.8 mm; Rad-score: 0.6246). Last row: A 48-year-old male with a lung adenocarcinoma in the right upper lobe (nodule size: 8.8 mm; Rad-score: 0.8375)

We then further investigated the relationships between radiomics features and lung adenocarcinoma group. Three core radiomics features as GLV (gray-level variance)-GLRLM (gray level run length matrix) -0.5-1-Lloyd-32 (OR = 1.993; 95% CI: 1.313–3.023), Entropy-GLCM (gray-level co-occurrence matrix)-2–0.8-Lloyd-64 (OR = 0.527; 95% CI: 0.344–0.805) and RLV (run-length variance)-GLRLM-2-2-Equal-64 (OR = 0.585; 95% CI: 0.397–0.860) were selected by multivariable logistic regression **(**Fig. 4**)**.

### Construction and validation of the radiomics nomogram

**Fig. 5:**
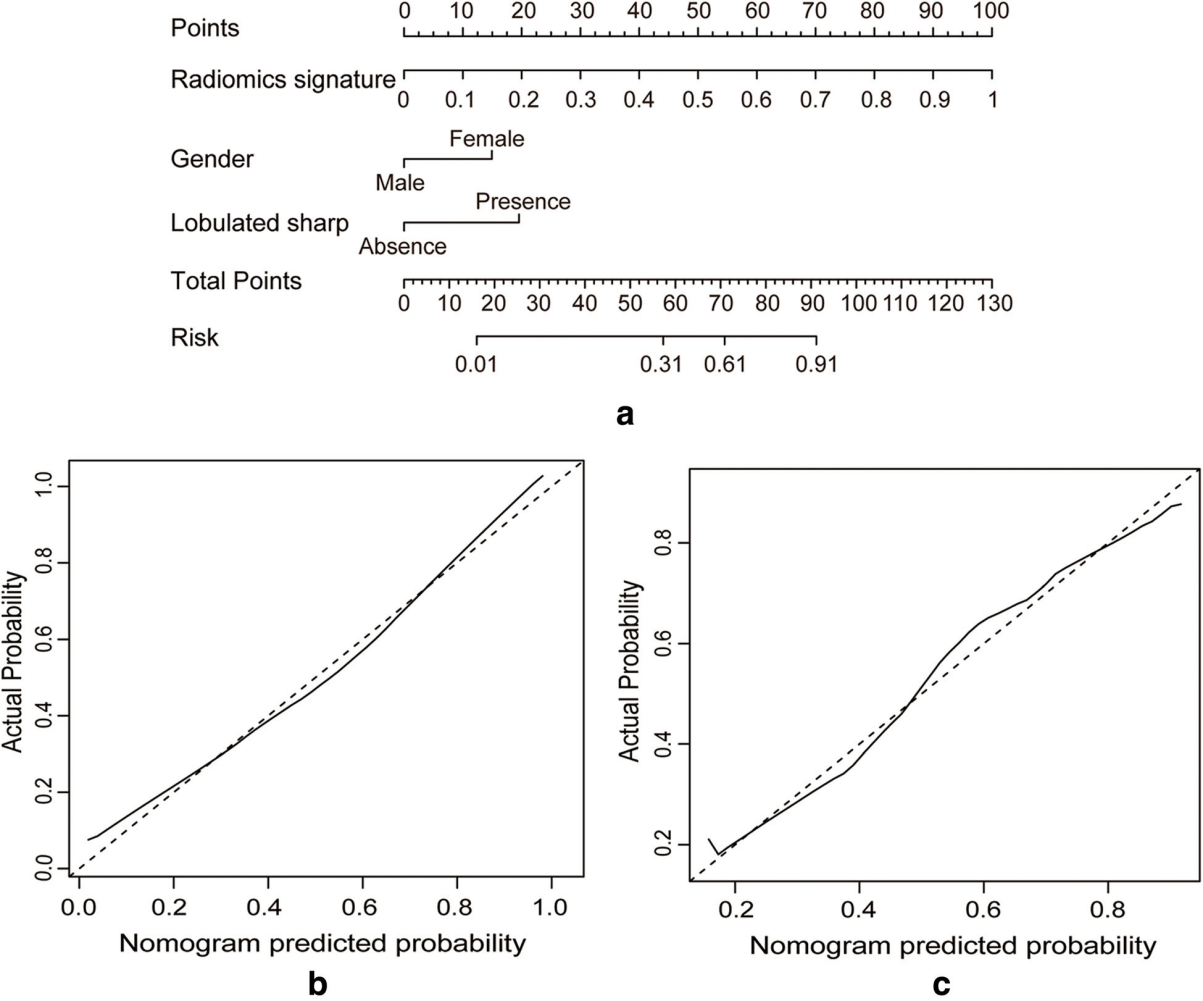
**a**) A radiomics nomogram incorporating clinical features and a radiomics signature was developed in the training dataset. Calibration curves of the radiomics nomogram being used in the training dataset (**b**) and external validation dataset (**c**). The y-axis represented the actual lung adenocarcinoma rate, and the x-axis represented the predicted lung adenocarcinoma possibility. The diagonal dashed line indicated the ideal prediction by a perfect model

According to the multivariate analysis, gender (OR = 0.255; 95% CI: 0.101–0.643), lobulated shape (OR = 6.029; 95% CI: 2.392–15.198) and radiomics signature (OR = 8.090; 95% CI: 3.772–17.354) were statistically significant independent differentiators of lung adenocarcinomas and granulomatous lesions, and they were used to develop the combined radiomics nomogram **(**Table 2**)**. Using the calibration curve, a marked connection between the predicted and actual data in the training set was confirmed (Fig. 5). The Hosmer–Lemeshow test yielded a non-significant statistical difference (*p* = 0.230).

**Table 2:** The parameters of the radiomics nomogram for lung adenocarcinomas and granulomatous lesions in patients with SCSNs of the training set

Intercept and Variable	β	Odds ratio (95% CI)	*P* value
Intercept	−0.115		0.744
Gender	−1.365	0.255 (0.101–0.643)	0.004*
Lobulated sharp	1.797	6.029 (2.392–15.198)	< 0.001*
Radiomics signature	2.091	8.090 (3.772–17.354)	< 0.001*

Note. β: the regression coefficient. *CI* Confidence interval. *p value < 0.05

*SCSNs* Sub-centimeter solid nodules

When subjective CT findings and the radiomics signature were combined, the AUC was increased to 0.885 (95% CI: 0.823–0.931), which was superior to the model generated with subjective findings alone in which the AUC = 0.762 (95% CI: 0.686–0.828), and the model created with the radiomics signature alone in which the AUC = 0.834 (95% CI: 0.764–0.889) **(**Table 3**)**.

**Table 3:** Predictive performance of subjective findings, radiomics signature and radiomics nomogram models for differentiating lung adenocarcinomas and granulomatous lesions in patients with SCSNs

	Training set (*n* = 150)			External validation set (*n* = 64)		
	Subjective findings model	Radiomics signature	Radiomics nomogram	Subjective findings model	Radiomics signature	Radiomics nomogram
AUC (95% CI)	0.762 (0.686–0.828)	0.834 (0.764–0.889)	0.885 (0.823–0.931)	0.619 (0.489–0.738)	0.798 (0.679–0.888)	0.808 (0.690–0.896)
Sensitivity	0.831 (64/77)	0.766 (59/77)	0.727 (56/77)	0.657 (23/35)	0.714 (25/35)	0.714 (25/35)
Specificity	0.603 (44/73)	0.781 (57/73)	0.904 (66/73)	0.621 (18/29)	0.828 (24/29)	0.828 (24/29)
Accuracy	0.720 (108/150)	0.773 (116/150)	0.813 (122/150)	0.641 (41/64)	0.766 (49/64)	0.766 (49/64)
PPV	0.688 (64/93)	0.787 (59/75)	0.889 (56/63)	0.676 (23/34)	0.833 (25/30)	0.833 (25/30)
NPV	0.772 (44/57)	0.760 (57/75)	0.759 (66/87)	0.600 (18/30)	0.706 (24/34)	0.706 (24/34)

Note. *CI* Confidence interval; *AUC* Area under curve; *NPV* Negative predictive value; *PPV* Positive predictive value. Numbers in the parentheses were used to calculate percentages. *SCSNs* Sub-centimeter solid nodules

**Fig. 6:**
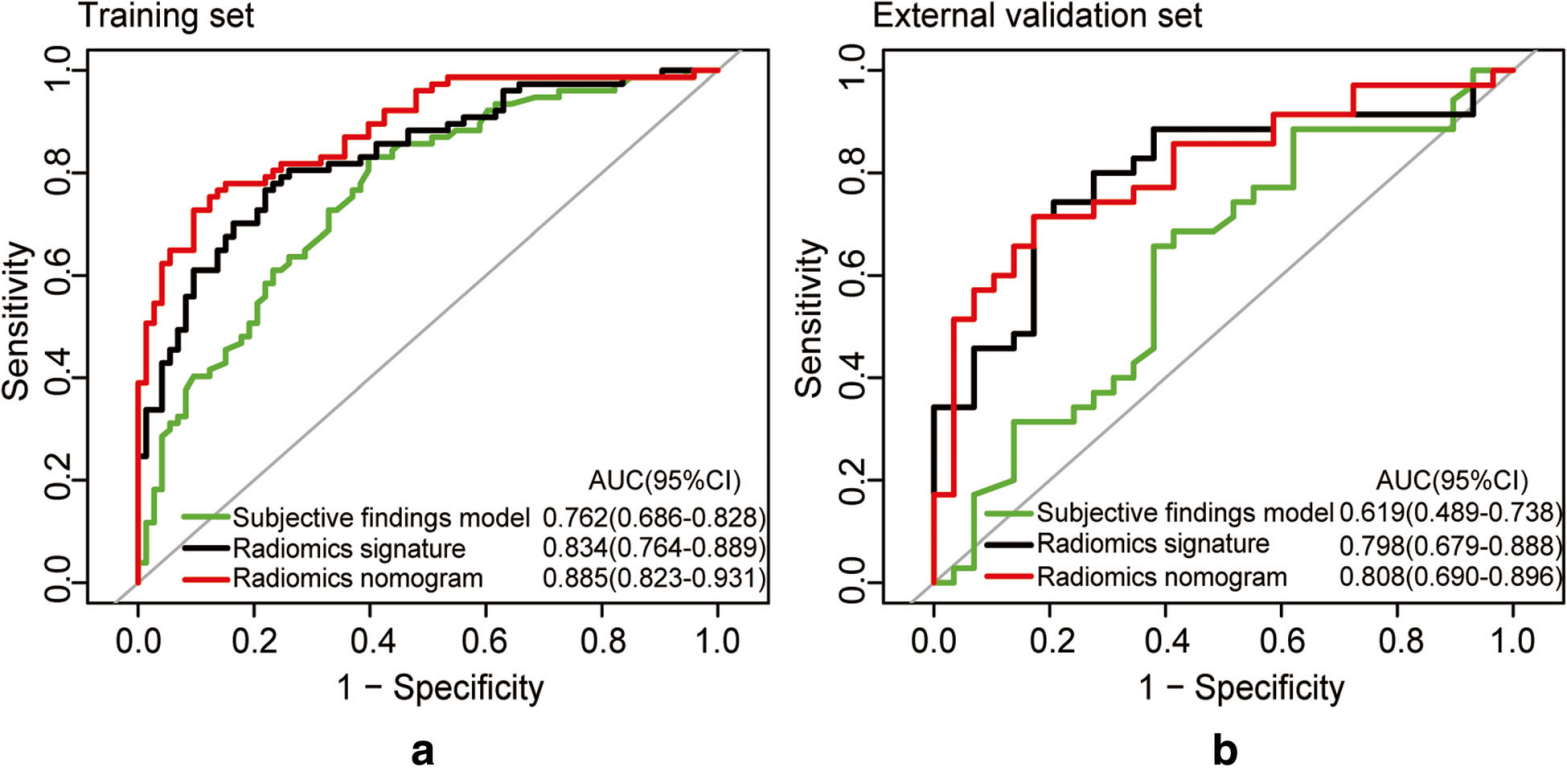
Receiver operating characteristic (ROC) curves of the 3 prediction models: subjective findings model, radiomics signature model, radiomics nomogram model. (**a**) Training dataset; (**b**) External validation dataset

With regard to validation, the radiomics nomogram exhibited the best discrimination ability in the external validation set (AUC = 0.808 (95% CI: 0.690–0.896); accuracy = 0.766; sensitivity = 0.714; specificity = 0.828) **(**Table 3**,** Fig. 6**)**. Significant differences between the subjective findings model and radiomics nomogram with respect to AUCs were found in the training set (Delong test: *p* < 0.001) and external validation set (Delong test: *p* = 0.004), respectively. The NRI (net reclassification index) indicated that the radiomics nomogram had significantly better predictive performance than the subjective findings model in both the training set (NRI = 0.804 (95% CI: 0.512–1.096); *p* < 0.001) and external validation set (NRI = 0.981 (95% CI: 0.575–1.388); *p* < 0.001). As shown in **Supplementary****A1****and Figure****S1****,** the stratified analysis showed that the performance of radiomic nomogram was not affected by gender, age, CT scan system, or CT image thickness (Delong tests: *p* > 0.05).

### Decision curve analysis

**Fig. 7:**
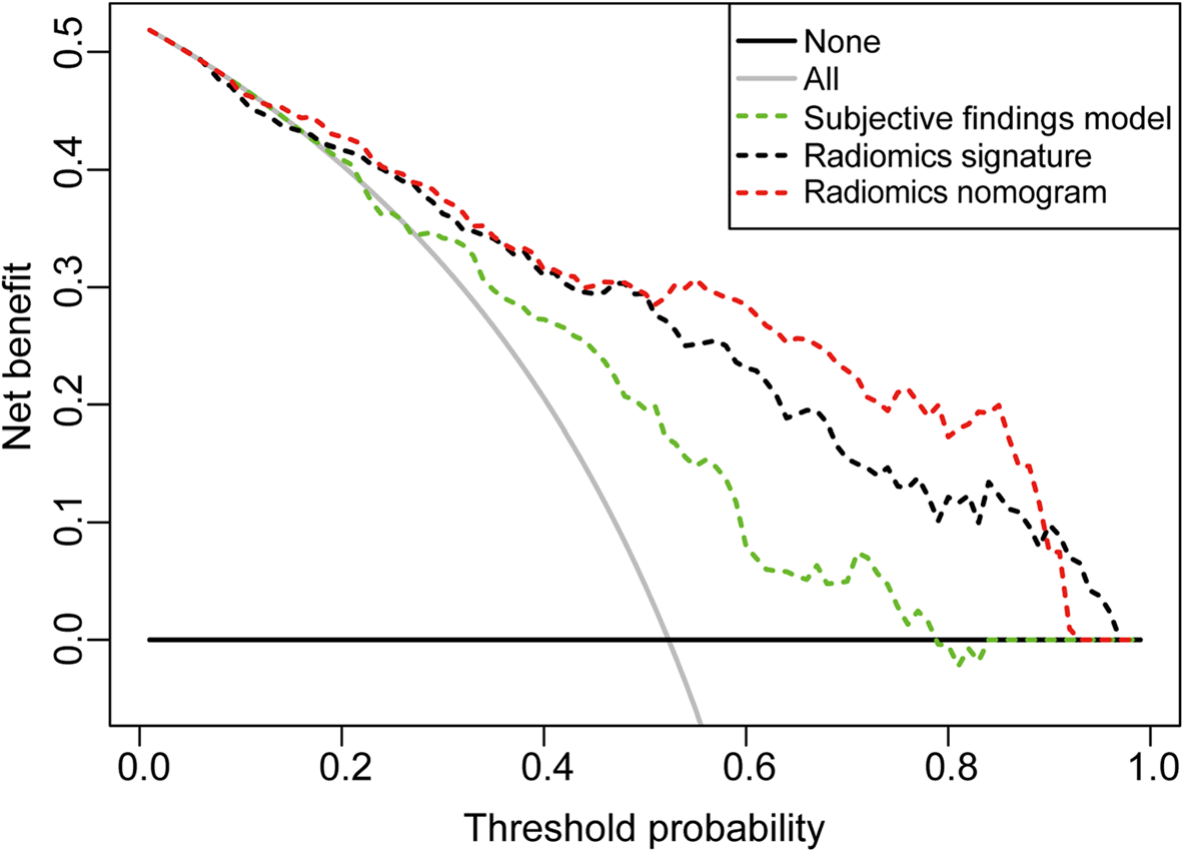
Decision curve analysis of the prediction models. The y-axis represented the net benefit. The dotted red line represented the radiomics nomogram model. The dotted green line represents the subjective findings model. The dotted black line represented the radiomics signature model. The solid gray line represented the assumption that all patients had lung adenocarcinomas. The solid black line represented the hypothesis that all patients had granulomatous lesions. The x-axis represented the threshold probability. The threshold probability was where the expected benefit of treatment was equal to the expected benefit of no treatment. The decision curve showed that the radiomics nomogram added more net benefit than the subjective findings model within the range of 0.13 to 0.98

The DCA for the radiomics nomogram was presented in Fig. 7**.** The decision curve showed that the radiomics nomogram added more net benefit than the subjective findings model in differentiating lung adenocarcinomas from granulomatous lesions within the range of the threshold probability of 0.13 to 0.98.

## Discussion

In the present study, a diagnostic radiomics nomogram incorporating a radiomics signature and clinical subjective characteristics was developed and validated for differentiating lung adenocarcinomas and granulomatous lesions in patients with SCSNs. DCA showed that the radiomics nomogram was clinically useful.

This study addresses a very important and significant diagnostic problem that differentiate benign from malignant lesions in lung SCSNs. In the lung cancer high-risk population it may not be suitable to wait 3 to 12 months to confirm whether a solid nodule is malignant or benign. This is especially true when the solid nodule has a higher probability of being an invasive adenocarcinoma, which is very different from sub-solid nodules which are slow-growing, have an indolent pathobiological behavior, and can be followed regularly. In addition, the differential diagnosis of solitary solid pulmonary nodules has proven to be more difficult than that of sub-solid nodules. Studies of patients who have received surgical resections have shown that more than 90% of sub-solid nodules can be malignant [35], while the malignancy rate of solid nodules ranges from 53 to 75% [36, 37]. This highlights the necessity of differentiating the nature of solid pulmonary nodules in an accurate and timely manner. Furthermore, SCSNs are not reliably characterized by PET/CT scanning, and biopsy is difficult to perform [12, 14]. Although an aggressive approach to resection will identify and treat more early-stage lung cancers, it can also subject patients with granulomatous lesions to the inherent risk of invasive surgery. On the other hand, a conservative approach of watchful waiting may result in the interval progression of otherwise curable malignancies.

Distinguishing small malignant nodules from the majority of benign nodules on chest CT images is particularly challenging because their morphologic characteristics are difficult to discern with visual inspection. The morphology of small nodules is less distinct, and management should be strongly influenced by the appearance of the nodule rather than by size alone. Radiologists typically risk stratify non-calcified pulmonary nodules by interpreting nodule characteristics such as location, attenuation, diameter, volume, and margins [38, 39]. Our findings showed that SCSN location is consistent with the natural history of lung cancer, as primary malignant nodules are commonly located in the upper lobes [40]. However, granulomatous lesions also common in the upper lobes, especially in the background of the high tuberculosis incidence in Asia area. Malignant nodules are more likely to have irregular, lobulated, or spiculated margins due to malignant cells spreading within the pulmonary interstitium and intra-tumor fibrosis. Benign nodules are associated with smooth, rounded borders, and exhibit a benign growth pattern. There is, however, a significant overlap between nodules with irregular margins seen in inflammatory/infectious conditions and smooth, rounded margins noted in up to 20% of primary lung cancers nodules [41]. This may be the reason why the subjective findings model exhibited poor to moderate performance in two datasets (AUC = 0.762, 0.619, respectively).

Radiomics is a developing field aimed at deriving automated quantitative imaging features from medical images that can predict tumor behavior non-invasively. The radiomics parameters of SCSNs could not be identified via visual inspection, but reflected heterogeneity quantitatively and reproducibility. The proposed radiomics features were categorized into non-textural and textural features based on statistical methods. The final predictive model demonstrated that GLV-GLRLM-0.5-1-Lloyd-32, RLV-GLRLM-2-2-Equal-64 and Entropy-GLCM-2-0.8-Lloyd-64 were significantly related to lung adenocarcinomas. GLV-GLRLM-0.5-1-Lloyd-32 was a measurement of the variance in the run gray level intensity. RLV-GLRLM-2-2-Equal-64 was a measurement of the variance in the run length. We hypothesize that this non-uniform intensity distribution of the run length reflects the heterogeneity of adenocarcinoma tumors. A higher RLV-GLRLM-2-2-Equal-64 value reflected a more complex texture pattern contained in the tumor volume, which suggested that adenocarcinomas were more heterogeneous. Entropy-GLCM-2-0.8-Lloyd-64 was a measurement of the randomness in neighborhood intensity values. This entropy-related radiomics feature was significantly higher in lung adenocarcinomas, presumably reflected the more complex and heterogeneous internal structure of malignant lesions when compared to granulomatous lesions.

Dennie et al. used texture analysis based on non-contrast CT to differentiate lung cancer and granulomas, and reported a sensitivity of 88% and specificity of 92% (AUC = 0.90 ± 0.06, *p* < 0.0001) [23]. However, their research sample only included 31 lung cancer patients and 24 granuloma patients, and their model was not validated on an independent external dataset. Yang et al. studied 302 patients with plain radiomics, and reported a sensitivity of 75.3% and specificity of 72.3% for differentiating solitary granuloma nodules from lung adenocarcinomas. Whereas, the diagnosis was not confirmed by surgical resection in all patients and only nodule size was used as the subjective CT finding [21]. Hawkins et al. demonstrated that radiomics could be applied to lung cancer CT screening CT to predict risk for lung cancer (accuracy = 80%, AUC = 0.75). Although the majority of study patients had solid nodules (*n* = 338), non-solid nodules (*n* = 58) and part-solid nodules (*n* = 41) were also included in the analysis. However, the CT image slice thickness in their study varied from 1.0 to 5.0 mm [17]. In the current study, the combined radiomics nomogram model demonstrated adequate discrimination in the training set (AUC = 0.885) and external validation set (AUC = 0.808), and demonstrated significantly improved predictive ability when compared with traditional subjective findings model (Delong test: *p* < 0.001, = 0.004, respectively).

We acknowledged several limitations to this study. First, the study design was retrospective, the sample size was relatively small and only one independent external validation center. Further studies should enroll more patients from multi-sites so that the radiomics nomogram model may be better trained and validated. Second, only surgically resected SCSNs that were histologically proven to be lung adenocarcinomas or granulomatous lesions were included. For this reason, our nodule samples might have been skewed toward morphologically more conspicuous or aggressive malignant nodules. In contrast, this inclusion criterion warranted a pathologically homogenous sample of nodules. Additionally, a wide range of CT scan systems with different scan techniques were used. These scan parameters may affect image quality parameters, such as resolution, noise, and the partial volume effect, which in turn can affect the quality of the extracted features [42]. However, to minimize these variabilities, all images included in the current study were thin-slice thickness CT images (0.625–1.5 mm). Moreover, image normalization and reproducibility studies were performed in the pre-processing phase, which is suitable for radiomics features analysis [43]. A stratified analysis on the version of CT scanners validated the generalizability of this nomogram.

## Conclusion

In conclusion, the radiomics signature identified from non-enhanced CT images may be useful for differentiating lung adenocarcinomas and granulomatous lesions in patients with SCSNs. The radiomics nomogram combining a radiomics signature and subjective findings maybe an effective tool for reducing overdiagnosis and overtreatment of SCSNs.

## Supplementary information

**Additional file 1: Supplementary A0**. Details for U-net based DL model. **Supplementary A1**. Stratified analysis of radiomic nomogram. **Supplementary Figure S1**. Radiomics nomogram for each subgroup stratified by (A) age, (B) gender, (C) CT scan system, and (D) CT image slice thickness, respectively. **Supplementary Table S1**. Radiomics score formulas

## Data Availability

The datasets used and/or analyzed during the current study are available from the corresponding author on reasonable request.

## Abbreviations

CT: Computed tomography
SCSNs: Sub-centimeter solid nodules
LASSO: Least absolute shrinkage and selection operator
AUC: Operating characteristic curve
PACS: Picture archiving and communication system
HU: Hounsfield units
WHO: World health organization.
VOI: Volume of interest.
ICC: Inter correlation coefficients.
KNN: K-nearest neighbor.
SVM: Support vector machine.
ROC: Receiver operating characteristics.
DCA: Decision curve analysis.
CI: Confidence interval.
