# Supplementary figures and images for: A CT-based radiomics nomogram for prediction of lung adenocarcinomas and granulomatous lesions in patient with solitary sub-centimeter solid nodules
Source: Cancer Imaging. 2020 Jul 8;20:45. doi: 10.1186/s40644-020-00320-3 (PMC7346427; doi:10.1186/s40644-020-00320-3)

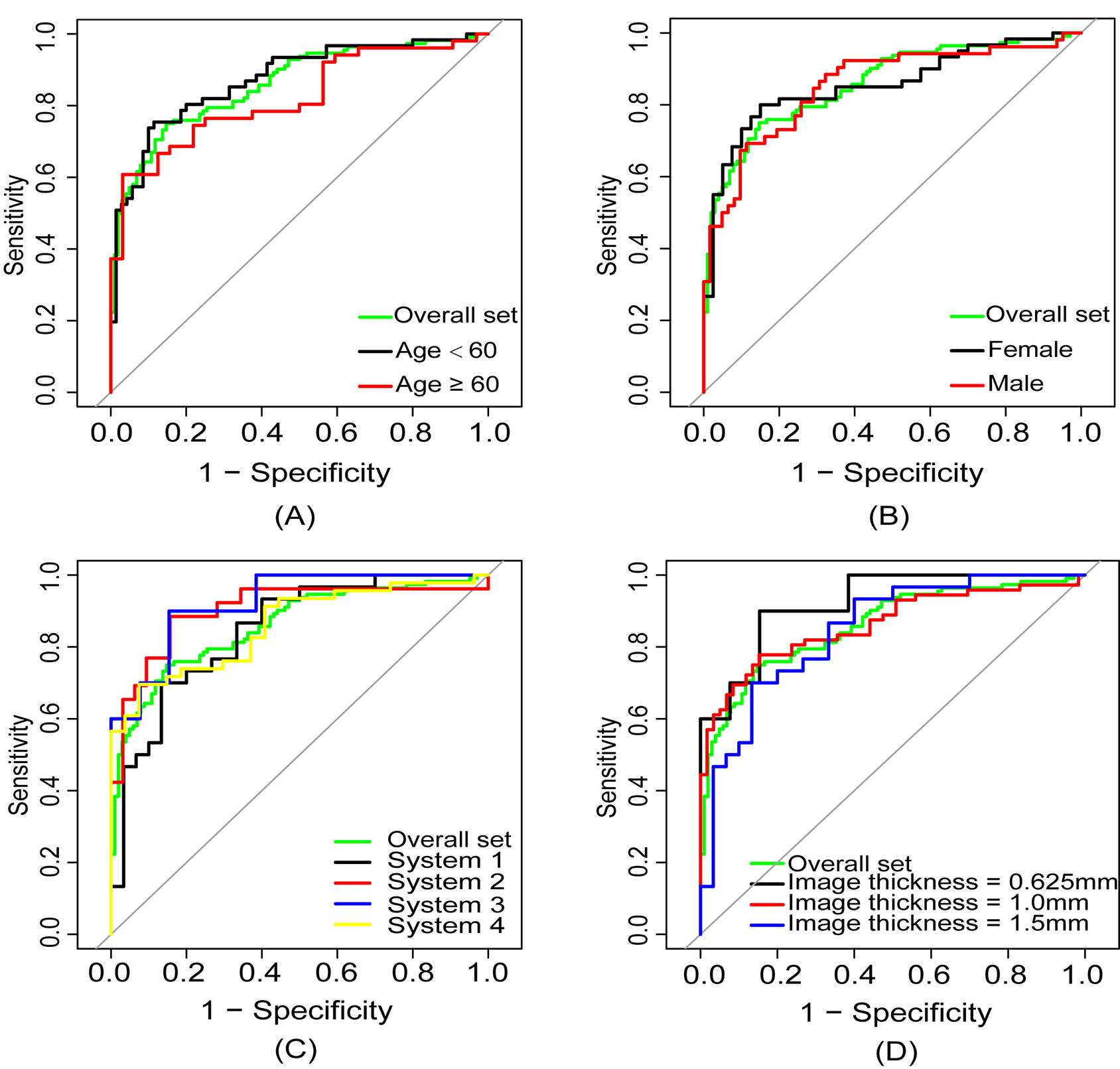

Supplement: Supplementary file 1 — Additional file 1: Supplementary A0. Details for U-net based DL model. Supplementary A1. Stratified analysis of radiomic nomogram. Supplementary Figure S1. Radiomics nomogram for each subgroup stratified by (A) age, (B) gender, (C) CT scan system, and (D) CT image slice thickness, respectively. Supplementary Table S1. Radiomics score formulas [file 40644_2020_320_MOESM1_ESM.zip › Revised Version of Supplementary Figure S1.tif]
