# Supplementary material for: A CT-based radiomics nomogram for prediction of lung adenocarcinomas and granulomatous lesions in patient with solitary sub-centimeter solid nodules
Source: Cancer Imaging. 2020 Jul 8;20:45. doi: 10.1186/s40644-020-00320-3 (PMC7346427; doi:10.1186/s40644-020-00320-3)
Supplement: Supplementary file 1 — Additional file 1: Supplementary A0. Details for U-net based DL model. Supplementary A1. Stratified analysis of radiomic nomogram. Supplementary Figure S1. Radiomics nomogram for each subgroup stratified by (A) age, (B) gender, (C) CT scan system, and (D) CT image slice thickness, respectively. Supplementary Table S1. Radiomics score formulas [file 40644_2020_320_MOESM1_ESM.zip › CAIG-D-19-00365 Revised Supplementary Data.doc]

**Supplementary Materials**

**Supplementary A0: Details for U-net based DL model**

Three segmentation performance evaluation indexes, including the true positive ratio (R_TP_), the false positive ratio (R_FP_) and the similarity ratio (R_S_) were utilized. The U-net DL model converged after 50 epochs of training. The average of *R_TP_*, *R_FP_*, and *D_S_* in external validation set were used to evaluate the segmentation results of the U-net based DL model. The results showed that U-net based DL model can segment tumor area more accurate and more robust with *R_TP_* = 0.91, *R_FP_* = 0.14, and *D_S_* = 0.88.

**Supplementary A1: Stratified analysis of radiomic nomogram**

The generalizability of a nomogram is always of great concern in terms of its application values. In order to test the generalization ability of our nomogram, we performed stratification analysis on the subgroups of age, gender, CT scan system and CT image slice thickness. We used the ROC curve and AUC to evaluate the performance of our radiomics nomogram on these subgroups. The results showed that the combined nomogram model was not influenced by these factors (all *p* > 0.05) (**Supplementary Figure S1**), indicating a good generalizability of the nomogram.

**1. Stratified analysis on age：**Patients were divided into two subgroups: age < 60 (131 cases) and age ≥ 60 (83 cases) with AUCs of 0.8719 and 0.8199. The AUC of overall set (214 cases) was 0.8590. (Delong test *p* value: 0.7488 and 0.4526 compared with the result on the overall set, respectively).

**2. Stratified analysis on gender：**Patients were divided into two subgroups: male (114 cases) and female (100 cases) with AUCs of 0.8536 and 0.8554. The AUC of overall set (214 cases) was 0.8590. (Delong test *p* value: 0.9042 and 0.9377 compared with the result on the overall set, respectively).

**3. Stratified analysis on CT scan system：**Patients were divided into four subgroups: scanned using Somatom Sensation 16-detector scanner (system 1，60 cases), Toshiba Aquilion 64-slice CT scanner (system 2, 58 cases), GE Discovery CT750 64-detector CT scanner (system 3, 23 cases) and Somatom Sensation dual-energy force (system 4, 73 cases) with AUCs of 0.8456, 0.9026, 0.9231 and 0.8591. The AUC of overall set (214 cases) was 0.8590. (Delong test *p* value: 0.8111, 0.4029, 0.2874 and 0. 9981 compared with the result on the overall set, respectively).

**4. Stratified analysis on CT image thickness:** Patients were divided into three subgroups: image thickness = 0.625 mm (23 cases), image thickness = 1.0 mm (131 cases) and image thickness = 1.5 mm (60 cases) with AUCs of 0.9231, 0.8616 and 0.8456. The AUC of overall set (214 cases) was 0.8590. (Delong test *p* value: 0.2874, 9494 and 0.8111 compared with the result on the overall set, respectively).

**Supplementary Figure S1.** Radiomics nomogram for each subgroup stratified by (A) age, (B) gender, (C) CT scan system, and (D) CT image slice thickness, respectively.

**Supplementary Table S1: Radiomics score formulas**

Ent-score = 0.0710

+ 0.1269* Busyness_NGTDM_0.5_1.5_Lloyd_8

+ 0.0586* SZHGE_GLSZM_2_1.2_Lloyd_32

- 0.0456* Variance_GLCM_1_1.5_Lloyd_8

- 0.0096* Contrast_GLCM_1.5_1.5_Equal_64

- 0.3471* SRLGE_GLRLM_0.67_0.8_Lloyd_16

- 0.0032* LRLGE_GLRLM_1.5_1.2_Lloyd_8

+ 0.0666* Contrast_NGTDM_0.67_2_Equal_32

+ 0.0115* LRHGE_GLRLM_2_0.8_Lloyd_64

+ 0.2952*GLV_GLRLM_0.5_ 1_Lloyd_32

+ 0.0727*RLV_GLRLM_0.5_1.5_Lloyd_16

+ 0.0168* RLV_GLRLM_1_1_Lloyd_64

- 0.0006* Variance_GLCM_2_1.5_Equal_64

+ 0.0156*SZE_GLSZM_0.5_1.5_Lloyd_16

- 0.1412* Entropy_GLCM_2_0.8_Lloyd_64

- 0.2089*Contrast_GLCM_2_1.5_Equal_64

+ 0.0461* SZHGE_GLSZM_2_1_Lloyd_8

- 0.0144*RLV_GLRLM_0.67_2_Equal_64

- 0.0487*HGRE_GLRLM_0.5_0.8_Equal_64

+ 0.0328*SZE_GLSZM_0.67_1.5_Lloyd_8

+ 0.0322*ZSV_GLSZM_1.5_1.2_Lloyd_16

+ 0.1431*SZLGE_GLSZM_2_1.2_Equal_16

- 0.2420*RLV_GLRLM_2_2_Equal_64

| **Groups** | **Features** | **Formulas** |
| --- | --- | --- |
| **GLCM** | Entropy | $-\sum_{i=1}^{N_{g}} \sum_{j=1}^{N_{g}} p(i,j)\log_{2}(p(i,j)+\varepsilon)$ |
|  | Contrast | $\sum_{i=1}^{N_{g}} \sum_{j=1}^{N_{g}} \left(i-j\right)^{2}p(i,j)$ |
|  | Variance | $\frac{1}{N_{g}\times N_{g}}\sum_{i=1}^{N_{g}} \sum_{j=1}^{N_{g}} [(i-\mu_{i})^{2}p(i,j)+(j-\mu_{j})^{2}p(i,j)]$ |
| **GLRLM** | RLV | $\sum_{j=1}^{L_{r}} \left(\sum_{i=1}^{N_{g}} p(i,j)\right)^{2}$ |
|  | HGRE | $\sum_{i=1}^{N_{g}} \sum_{j=1}^{L_{r}} i^{2}p(i,j)$ |
|  | GLV | $\sum_{i=1}^{N_{g}} \sum_{j=1}^{L_{r}} \frac{1}{N_{g}+L_{r}}\left(ip(i,j)\text{-}\mu_{i}\right)^{2}$ |
|  | LRHGE | $\sum_{i=1}^{N_{g}} \sum_{j=1}^{L_{r}} i^{2}j^{2}p(i,j)$ |
|  | LRLGE | $\sum_{i=1}^{N_{g}} \sum_{j=1}^{L_{r}} \frac{j^{2}p(i,j)}{i^{2}}$ |
|  | SRLGE | $\sum_{i=1}^{N_{g}} \sum_{j=1}^{L_{r}} \frac{p(i,j)}{i^{2}j^{2}}$ |
| **GLSZM** | SZE | $\sum_{i=1}^{N_{g}} \sum_{j=1}^{L_{z}} \frac{p(i,j)}{j^{2}}$ |
|  | SZHGE | $\sum_{i=1}^{N_{g}} \sum_{j=1}^{L_{z}} \frac{i^{2}p(i,j)}{j^{2}}$ |
|  | SZLGE | $\sum_{i=1}^{N_{g}} \sum_{j=1}^{L_{z}} \frac{p(i,j)}{i^{2}j^{2}}$ |
|  | ZSV | $\sum_{i=1}^{N_{g}} \sum_{j=1}^{L_{z}} \frac{1}{N_{g}+L_{z}}\left(jp(i,j)\text{-}\mu_{j}\right)^{2}$ |
| **NGTDM** | Busyness | $\frac{\sum_{i=1}^{N_{g}} n_{i}P(i)}{\sum_{i=1}^{N_{g}} \sum_{j=1}^{N_{g}} (in_{i}-jn_{j})},n_{i}\neq0,n_{j}\neq0$ |
|  | Contrast | $\begin{matrix} \left(\frac{1}{N_{g,p}(N_{g,p}-1)}\sum_{i=1}^{N_{g}} \sum_{j=1}^{N_{g}} p_{i}p_{j}(i-j)^{2}\right)\left(\frac{1}{N_{v,p}}\sum_{i=1}^{N_{g}} s_{i}\right), \\ where p_{i}\neq0,p_{j}\neq0 \end{matrix}$ |

Notes:

GLCM: Gray-level co-occurrence matrix.

GLRLM: Gray Level Run Length Matrix.

GLSZM: Gray Level Size Zone Matrix.

NGTDM: Neighboring Gray Tone Difference Matrix.

**Part 1: Gray-level co-occurrence matrix (GLCM)**

A: Entropy

$$Entropy=-\sum_{i=1}^{N_{g}} \sum_{j=1}^{N_{g}} p(i,j)\log_{2}(p(i,j)+\varepsilon)$$

B: Contrast

$$Contrast=\sum_{i=1}^{N_{g}} \sum_{j=1}^{N_{g}} (i-j)^{2}p(i,j)$$

C: Variance

$$Variance=\frac{1}{N_{g}\times N_{g}}\sum_{i=1}^{N_{g}} \sum_{j=1}^{N_{g}} [(i-\mu_{i})^{2}p(i,j)+(j-\mu_{j})^{2}p(i,j)]$$

Where $N_{g}$is the number of discrete intensity levels in VOI. $p(i,j)$ represents the number of times that a voxel with gray level i and gray level j are adjacent to each other in VOI. $\varepsilon$is an arbitrarily small positive number ($\approx2.2\times10^{-16}$).

**Part 2: Gray Level Run Length Matrix (GLRLM)**

A: Run-Length Nonuniformity (RLV)

$$RLV =\sum_{j=1}^{L_{r}} \left(\sum_{i=1}^{N_{g}} p(i,j)\right)^{2}$$

B: High Gray Level Run Emphasis (HGRE)

$$HGRE =\sum_{i=1}^{N_{g}} \sum_{j=1}^{L_{r}} i^{2}p(i,j)$$

C: Gray Level Variance (GLV)

$$GLV=\frac{1}{N_{g}\times L_{r}}\sum_{i=1}^{N_{g}} \sum_{j=1}^{L_{r}} (ip(i,j)-\mu_{i})^{2}$$

D: Long Run High Gray Level Emphasis (LRHGE)

$$LRHGE=\sum_{i=1}^{N_{g}} \sum_{j=1}^{L_{r}} i^{2}j^{2}p(i,j)$$

E: Long Run Low Gray Level Emphasis (LRLGE)

$$LRLGE=\sum_{i=1}^{N_{g}} \sum_{j=1}^{L_{r}} \frac{j^{2}p(i,j)}{i^{2}}$$

F: Short Run Low Gray Level Emphasis (SRLGE)

$$SRLGR=\sum_{i=1}^{N_{g}} \sum_{j=1}^{L_{r}} \frac{p(i,j)}{i^{2}j^{2}}$$

Where $N_{g}$ is the number of discreet intensity values in VOI. $L_{r}$is the number of discreet run lengths in VOI. $p(i,j)$represents the number of runs of gray-level $i$ and of length $j$ in VOI.

**Part 3: Gray Level Size Zone Matrix (GLSZM)**

A: Small Zone Emphasis (SZE)

$$SZE=\sum_{i=1}^{N_{g}} \sum_{j=1}^{L_{z}} \frac{p(i,j)}{j^{2}}$$

B: Small Run High Gray Level Emphasis (SZHGE)

$$SZHGE=\sum_{i=1}^{N_{g}} \sum_{j=1}^{L_{z}} \frac{i^{2}p(i,j)}{j^{2}}$$

C: Small Zone Low Gray Level Emphasis (SZLGE)

$$SZLGE=\sum_{i=1}^{N_{g}} \sum_{j=1}^{L_{z}} \frac{p(i,j)}{i^{2}j^{2}}$$

D: Zone size variance (ZSV)

$$ZSV=\frac{1}{N_{g}\times L_{z}}\sum_{i=1}^{N_{g}} \sum_{j=1}^{L_{z}} (jp(i,j)-\mu_{j})^{2}$$

Where $N_{g}$ is the number of discreet intensity values in VOI. $L_{z}$is the number of discreet zone sizes in VOI. $p(i,j)$represents the number of 3D zones of gray-levels $i$ and of size $j$ in VOI.

**Part 4: Neighboring Gray Tone Difference Matrix (NGTDM)**

A: Busyness

$$busyness=\frac{\sum_{i=1}^{N_{g}} n_{i}P(i)}{\sum_{i=1}^{N_{g}} \sum_{j=1}^{N_{g}} (in_{i}-jn_{j})},n_{i}\neq0,n_{j}\neq0$$

B: Contrast

$$Contrast=\left(\frac{1}{N_{g,p}(N_{g,p}-1)}\sum_{i=1}^{N_{g}} \sum_{j=1}^{N_{g}} p_{i}p_{j}(i-j)^{2}\right)\left(\frac{1}{N_{v,p}}\sum_{i=1}^{N_{g}} s_{i}\right), where p_{i}\neq0,p_{j}\neq0$$

Where $N_{g}$ is the number of discreet gray levels. $N_{v,p}$ is the total number of voxels in $X_{gl}$ and equal to$\sum n_{i}$ (i.e. the number of voxels with a valid region; at least 1 neighbor). $N_{v,p}\leq N_{p}$, where $N_{p}$ is the total number of voxels in the ROI. $s_{i}$ is the sum of absolute differences for gray level $i$. $p_{i}$ is the gray level probability.
